# Supplementary material for: Applying linear programming in evaluating employees in higher education: A case study
Source: PLoS One. 2025 Jan 17;20(1):e0310183. doi: 10.1371/journal.pone.0310183 (PMC11741587; doi:10.1371/journal.pone.0310183)
Supplement: Attachment — Submitted filename: Response to Reviewers.pdf [file pone.0310183.s001.pdf]

Response to Reviewers

Manuscript ID: PONE-D-24-15475

Title: Applying the linear programming in the evaluation of employees in the higher education sector-Case study

Dear Dr. Claudia Noemi González Brambila, Ph.D., Academic Editor, PLOS ONE,

We appreciate the valuable feedback provided by the reviewers. We have addressed each of their comments and made the necessary revisions to enhance the manuscript. Below is a detailed response to the reviewers' comments:

Reviewer #1:

1. **Enumerate all 7 stages, not just 5.**
    - We have revised the manuscript to explicitly enumerate all seven stages of the proposed model. The stages are now clearly listed and explained in the methodology section.
2. **Justification or demonstration of the linearity of the problem.**
    - A justification for the linearity of the problem has been added to the methodology section, explaining why linear programming is appropriate for this study.
3. **Specific advantages and disadvantages related to the project.**
    - We have included a discussion on how the advantages and disadvantages of linear programming specifically relate to the project.
4. **Clear problem statement.**
    - The problem statement has been clarified at the beginning of the paper, emphasizing that no new professors are being hired and the selection is for a new program.
5. **Diagnostic variables.**
    - The diagnostic variables have been corrected to ensure there are no duplicates, and the subsets are clearly distinguished.
6. **Weak Stage 1 and unexplained Stage 6.**
    - Stage 1 has been significantly expanded to include detailed steps involved in determining the availability of research and teaching staff. Stage 6 has been elaborated to provide a clearer explanation of the development of the mathematical model.
7. **Correction of Table references.**

- All table references have been checked and corrected to ensure they are correctly used in the corresponding sections.

8. **Title of the paper.**
   - The title has been modified to better reflect the content: “Applying Linear Programming in Evaluating Employees in Higher Education: A Case Study”.

Reviewer #2:

1. **Page 2, second paragraph, first sentence. Change to higher education.**

   The sentence has been revised to: “In the higher education sector, HRM practices focus on evaluating and enhancing the knowledge and abilities of human resources.”

2. **Page 2, middle third paragraph. "Once, is related...." I don't understand that sentence. Also, "Many research...." probably should be much research.**
   - The sentence has been clarified and revised to: “In the higher education sector, HRM practices focus on evaluating and enhancing the knowledge and abilities of human resources. Much research shows that having a proper HRM system directly impacts employee performance by influencing their attitudes and behavior, leading to increased productivity and higher organizational performance.”

3. **Page 3, Last paragraph, second sentence, the end should be "are as follows:"**
   - The sentence has been corrected to end with “are as follows:”.

4. **Address self-reporting limitations and selection of criteria.**
   - A significant limitation related to the reliance on self-reporting survey data has been added to the limitations section. Additionally, the importance of selecting criteria that align with both management and student priorities has been discussed, and the model's customization potential has been highlighted.
